# Altered temporal sensitivity in obesity is linked to pro-inflammatory state

**DOI:** 10.1038/s41598-019-51660-5

**Published:** 2019-10-29

**Authors:** Federica Scarpina, Paolo Marzullo, Stefania Mai, Alessandro Mauro, Massimo Scacchi, Marcello Costantini

**Affiliations:** 1 Istituto Auxologico Italiano, I.R.C.C.S., Ospedale San Giuseppe, Piancavallo, Italy; 2 Department of Translational Medicine, Università del Piemonte Orientale ‘A. Avogadro’, Novara, Italy; 3 “Rita Levi Montalcini” Department of Neuroscience, University of Turin, Turin, Italy; 4 Department of Clinical Sciences and Community Health, University of Milan, Milan, Italy; 5 Department of Psychological, Health, and Territorial Sciences, “G. d’Annunzio” University of Chieti-Pescara, Chieti, Italy

**Keywords:** Perception, Human behaviour

## Abstract

Temporal sensitivity to multisensory stimuli has been shown to be reduced in obesity. We sought to investigate the possible role of the pro-inflammatory state on such alteration, considering the effect of the expression of markers, such as leptin and IL6, which are notably high in obesity. The performance of 15 male individuals affected by obesity and 15 normal-weight males was compared using two audiovisual temporal tasks, namely simultaneity judgment and temporal order judgment. Analyses of serum levels of inflammatory markers of leptin and IL6, and of neurotrophic factors of BDNF and S100SB were quantified. At the behavioral level we confirmed previous evidence showing poorer temporal sensitivity in obesity compared to normal-weight participants. Furthermore, leptin, that is a cytokine overexpressed in obesity, represented the best predictor of behavioral differences between groups in both tasks. The hypothesis we put forward is that the immune system, rather than overall cerebral dysfunction, might contribute to explain the altered temporal sensitivity in obesity. The present finding is discussed within the context of the role of cytokines on the brain mechanisms supporting temporal sensitivity.

## Introduction

The brain mechanism through which two or more different sensory input, when occur at the same time and place, are coordinate together to create an unified and coherent internal representation of the world, is known as *multisensory integration*^1^. It has a substantial survival value for humans: the construction of a coherent multimodal representation of the external world allows humans to take advantage of the redundancies and complementarities provided by multiple sensory modalities^2^. Thus, considering the large impact of this process on our perception, it is not surprising to observe a growing interest in studying multisensory integration, and under what circumstances this process takes place or defects. Indeed, multisensory integration difficulties has been recognized in several clinical conditions^3–5^; for example, our group recently provided evidence about aberrant temporal multisensory process in obesity^6^. Specifically, the behavioral measure of the audiovisual sensory integration, that it the *temporal binding window*, (i.e. *“the epoch of time within which stimuli from different modalities is likely to be integrated and perceptually bound”* according to the definition provided by Wallace and Stevenson (2014)^7^, resulted to be markedly different in the participants affected by obesity as compared to healthy-weight controls; our result mirrored what found previously by Wan and colleagues (2014)^8^ about the integration of audio-vibrotactile sensory input in overweight individuals.

A growing body of evidence suggests that the correct functioning of cerebral mechanisms, such as the *synchronized network oscillations*^9^ (i.e. the rhythmic or repetitive electrical activity generated spontaneously and in response to stimuli by neural tissue in the central nervous system; it is one of the neural mechanisms implicated in multisensory integration process^10–13^), is related to the immune systems^14^, which act on the central nervous system through chemical messengers^15^. In fact, as neural cells have receptors for cytokines, immune cells have receptors for various neurotransmitters^16^ involved in brain activity^14^. For example, the well-known γ-aminobutyric acid (GABA), that is the principal inhibitory neurotransmitter in the central nervous system^17^, is essential for the efficient brain functioning, mediating neuronal activity, information processing and plasticity, and the synchronized network oscillations^14^. Glutamate^18^ and GABA^19^ were found to predict individual differences in multisensory processing in healthy individuals: these components have inhibitory and exhibitory neuromodulatory effects, regulating the brain’s response to sensory perception^20^. Interestingly, as suggested by multiple research in animals and humans, as well as in pathological and clinical conditions, both glutamate^21^ and GABA^22^ are strictly related to inflammation: they can be considered as immunomodulators.

As in our knowledge, there is very few recent experimental evidence on the relationship between temporal sensitivity and inflammation. Focusing on obesity, as previously stated, our recent study^6^ and Wan and colleagues (2014)^8^’s work provided evidence about aberrant temporal multisensory process in this clinical condition. On the other hand, few research showed a correlation between resting state network dynamics and obesity^23^, and specifically an increased resting-state functional connectivity in severe obese adolescents^24^. How might these two different experimental evidences - the first one about an alteration of multisensory integration, the secondabout an alteration of cerebral activity in obesity be jointed? What might be the link between the functional cognitive process (i.e. multisensory integration) and cerebral activity? The turning point might be to look at obesity as not just a clinical condition caused by the accumulation of fat, but as a more complex condition in which metabolic dysfunction affects central nervous system. Specifically, it is now, even though not fully^25^ established, that obesity is associated with chronic low-grade systemic inflammation^26,27^; indeed the adipose tissue secretes a range of bioactive peptides and proteins^28^, included pro-inflammatory cytokines, with knock-on effects on other complex metabolic processes. Cytokines are a type of regulators of host responses to infection, immune responses, inflammation, and trauma^29^. When the cytokines actions result in a worsening of the disease, they are defined as pro-inflammatory; when they are overexpressed, as in in obesity^28^, they define a state of chronic systemic inflammation, meaning a prolonged condition in which the entire body is active to react to an illness. One the most studied product of the adipose tissue is leptin: it mediates the relationship between environment (intended as availability of nutrients), metabolism, and immune responses^30^. Another known product of the fat is the interleukin 6 (IL-6); the expression of this cytokine increases with adiposity^31^ with an important role in the expression of the other cytokines^32^. Interestingly, even though cytokines are produced at the level of adipose tissue (i.e. a peripheral inflammation), their negative consequences in obesity can be observed far away from the production site (i.e. systemic inflammation), influencing behavior as well as brain development and functions^33,34^. Brain is, indeed, a key target of the chronic low-grade inflammation, which is a risk factor for neuroinflammation and anatomical alterations^25,35–38^. The mechanisms according to inflammatory state might alter anatomically and functionally brain activity is not clear yet (see Miller and Spencer (2014)^25^, for a review). However, the existing relationship between and brain functioning, and the evidence showing altered brain functioning in obesity, pose the question whether inflammation might represent a link between obesity and altered brain mechanisms^25^, such as temporal sensitivity to multisensory stimuli.

Moreover, factors that are generally considered as indices of cerebral plasticity are found to be altered in obesity, in absence of neurological disease: this is the case for brain-derived neurotrophic factor (BDNF)^39,40^ and S100 calcium binding protein B (S100B)^41,42^. In details, BDNF plays a prominent role in the survival, growth, and maintenance of neurons during development^43,44^ in the adult age^45^. S100B, that is a member of the S100 protein family, is commonly used as a parameter of glial activation and/or death in different disorders of the central nervous system; since its role in normal cerebral development and recovery after injury, it was suggest to be a potential neurobiochemical marker of neurological disorders^46^.

Thus, given the tight link between the brain and the immune system^15^, we tested the hypothesis that inflammatory state in obesity might be related to poorer temporal sensitivity^6^. In particular, we address the question as to whether circulating levels of pro-inflammatory markers, such as leptin and IL-6, or markers of cerebral plasticity, such as BDND and S100B, are related to the temporal sensitivity in obesity, through a cross-sectional study. To this aim, the neurocognitive behavior of a sample of otherwise healthy obese men was tested by two experimental tasks assessing temporal sensitivity to multisensory stimuli^6^ compared to that obtained in a sample of control individuals with normal weight.

Blood levels of leptin, IL-6, BDNF and S100B were measured at the time of the testing in order to investigate the potential association with the pro-inflammatory pattern and its relation with neurotrophins. Pro-inflammatory cytokines production increases in response to acute psychological stress in humans^47,48^, suggesting a relationship between stress and immune functions^49,50^. Since this relationship appears to be critical in obesity^51–53^, we also measured the level of cortisol in our population sample.

## Results

### Demographic and clinical data

 The participants with obesity and the healthy-weight participants were comparable in terms of *Age* (t(28) = 1.26; p = 0.21; d = 0.61; 95% CI [2; 8.4]) and years of *Education* (t(28) = 1.94; p = 0.062; d = 0.63; 95% CI [0.14; 5.34]). Data are reported in Table 1. As expected, participants affected by obesity reported a significantly higher *BMI* than normal-weight participants (t(28) = 18.68; p < 0.001; d = 6.83; 95% CI [18.21; 22.69]); specifically for participants with obesity the mean value was higher than 44, suggesting that this condition can be classified as “*morbidly obese stage III*”^54^. No differences were observed when comparing Beck Depression Inventory^55,56^ scores between the two groups (t(28) = 1.88; p = 0.07; d = 0.69; 95% CI [0.21; 5.28]). Moreover, both groups reported the same level of daytime sleepiness, as suggested by the score at the Epworth Sleepiness Scale^57^ (t(28) = 1.48; p = 0.14; d = 0.54; 95% CI [0.83; 5.23]).

**Table 1:** Demographic and clinical data for the participants with obesity and the normal-weight participants.

	Participants with obesity	Healthy-weight participants	p-value
Age	34 (6)	30 (7)	0.21
Education	13 (4)	15 (2)	0.06
BMI	44.24 (3.7)	23.7 (2)	<0.001*
BDI score	6.4 (4.2)	3.86 (3.06)	0.07
ESS score	6.6 (4.98)	4.4 (2.84)	0.14
Leptin	42.77 (21.84)	2.92 (2.04)	<0.001*
IL6	1.64 (0.79)	0.49 (0.29)	<0.001*
BDNF	17.82 (3.56)	13.83 (3.02)	0.003*
S100B	23.24 (12.39)	20.05 (17.53)	0.57
Cortisol	12.84 (4.33)	14.39 (3.63)	0.3

Means and standard deviations (in brackets) are reported. Age in years is reported; Education is reported as years of school attended; BMI = body mass index express in units of kg/m^2^. BDI stands for the Beck Depression Inventory; ESS for the Epworth Sleepiness Scale. *Identifies a *significant difference* between the two groups.

### Serum markers

Means and standard deviations of the serum marker levels are reported in Table 1. A significant difference emerged between groups in the serum levels of *leptin* (Levene’s test F = 27.82; p < 0.001; t (14.24) = 7.036; p < 0.001: d = 2.56; 95% CI [27.72; 51.98]), as well as *IL6* (Levene’s test F = 12.17; p = 0.002; t (17.84) = 5.28; p < 0.001; d = 1.93; 95% CI [0.69; 1.61]) and *BDNF* (t(28) = 7.036; p = 0.003; d = 1.2; 95% CI [1.5; 6.45]). Instead, no differences were found for *S100B* (Levene’s test F = 4.59; p = 0.041; t(25.19) = 0.57; p = 0.57; 95% CI [8.17; 14.54]) and *cortisol* (t(27) = 1.03; p = 0.3; 95% CI [1.51; 4.6]).

### Experimental tasks

**Figure 1:**
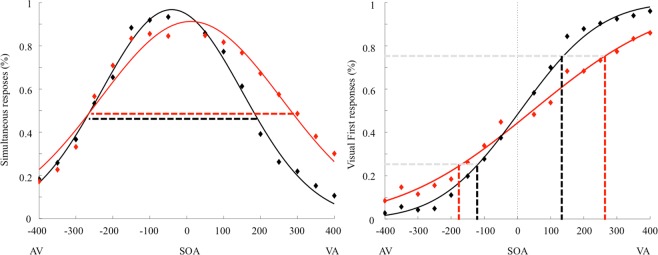
Left plot: Group mean TBW defined using the Simultaneity Judgment (SJ) task. Right plot: Group mean JND values defined using the Temporal Order Judgment (TOJ) task. Red curves represent obese participants, black curves represent healthy-weight controls. Symbols represent the raw, unfitted data.

Regarding the SJ, we evaluated the goodness of fit of our mathematical model: considering the data relative to the participants with obesity, the mean R^2^ was 0.86, while for the normal-weight group it was 0.93. The *Temporal Binding Windows* (TBW) values violated normality (Shapiro-Wilk test, p = 0.046), hence data were log-transformed to obtain a normal distribution. According to the independent sample t-test, participants with obesity (Mean = 343 ms; SD = 84 ms) showed a wider TBW respect to the normal-weight group (Mean = 277 ms; SD = 80 ms) (t(28) = 2.16; p = 0.039; d = 0.8; 95% CI [− 126.67; −3.58]) (Fig. 1, left plot), confirming our previous results^6^.

**Figure 2:**
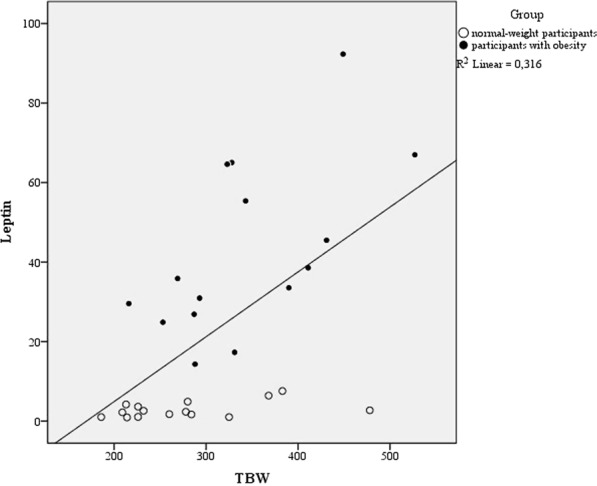
The relationship between the TBW (x-axys) defined using the Simultaneity Judgment (SJ) Task and the level of leptin (y axys) split for the two groups (filled circle: participants with obesity; empty circle: normal-weight participants).

According to the linear regression analysis, a significant regression was found [F(5, 23) = 3.23; p = 0.023] with an R^2^ of 0.41. A low level of multicollinearity was present (VIF = 2.5 for leptin, 2.17 for IL6, 1.1 for BDNF, 1.15 for S100B and 1.36 for cortisol). Leptin was the only significant predictor of the TBW [B = 2.097; p = 0.023] (Fig. 2); no other predictors were significant [*IL6* B = −13.69; p = 0.58; *BDNF* B = −4.39; p = 0.25; *S100B* B = 1.12; p = 0.27; *Cortisol* B = −3; p = 0.46].

**Figure 3:**
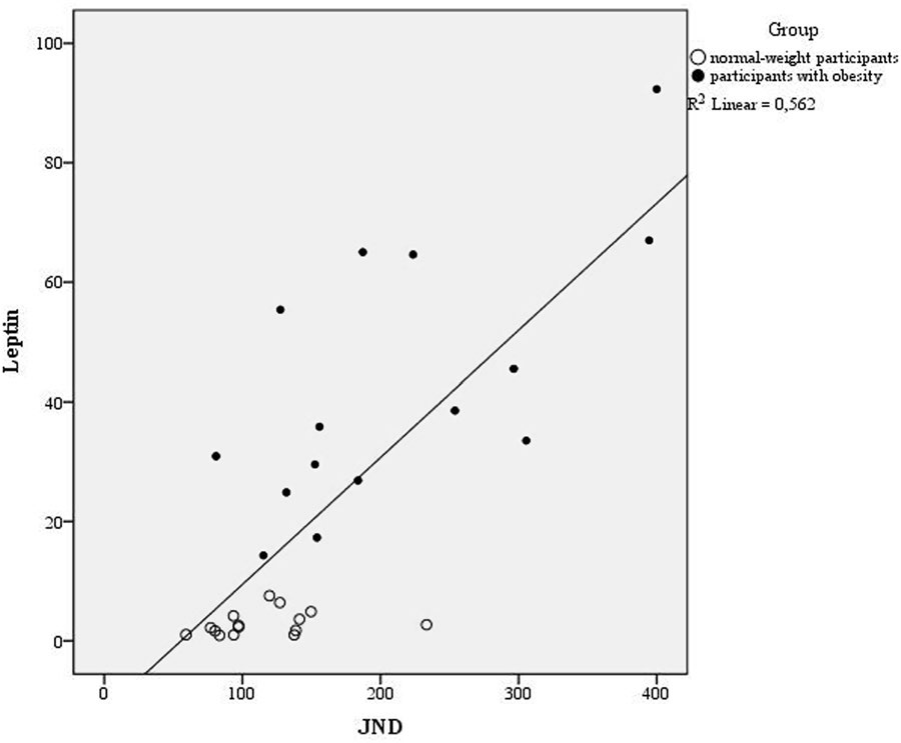
The relationship between the JND (x-axys) using the Temporal Order Judgment (TOJ) task. and the level of leptin (y axys) split for the two groups (filled circle: participants with obesity; empty circle: normal-weight participants).

Regarding TOJ, we evaluated the goodness of fit: for the data relative to the participants with obesity, we observed a mean R^2^ of 0.84, while in the normal-weight group it was 0.96. The *Just Noticeable Difference* (JND) values violated normality (Shapiro-Wilk test, p < 0.001), hence again data were log-transformed to obtain a normal distribution. According to the independent sample t-test, the results showed larger JND in participants with obesity (Mean = 210 ms; SD = 99) compared to healthy-weight controls (Mean = 115 ms; SD = 42), t(28) = 3.76; p = 0.001; d = 1.24; 95% CI [−0.37; −0.11] (Fig. 1, right plot). This result is in line with our previous report^23^. According to the linear regression analysis, a significant regression equation was found [F(5, 23) = 8.34 p < 0.001] with an R^2^ of 0.64. *Leptin* [B = 3.29; p < 0.001] was the only significant predictor also for the JND (Fig. 3); no other predictors were significant [*IL6* B = −32.77; p = 0.1; *BDNF* B = 0.15; p = 0.96; *S100B* B = 1.16; p = 0.15; *Cortisol* B = 0.14; p = 0.96].

Overall, we confirmed that individuals with obesity showed a wider TBW in SJ and a higher JND in TOJ respect to the healthy-weight controls, suggesting an alteration in the process of integration of multiple sensory stimuli. The behavior was predicted by the level of leptin measured in the serum in both tasks, as indicated by results in the linear regression analyses.

## Discussion

In the present work, we searched for an alteration of temporal sensitivity to multisensory stimuli in individuals affected by obesity. Moreover, we extend our investigation to test a potential relationship linking neurocognitive behavior to blood-based markers for pro-inflammatory state (leptin and IL6) and to those neurotrophic factors considered sensitive biochemical markers of cerebral injury (BDNF and S100B).

Focusing on the behavioral task, we found that temporal sensitivity to multisensory stimuli was markedly wider in participants affected by obesity compared to healthy-weight participants. This result is in line with our previous results^6^ and also it mirrors what reported by Wan and colleagues (2000)^8^. Furthermore, in the present study, we showed that the difficulties of individuals affected by obesity seem to be related to higher levels of pro-inflammatory markers, instead of indexes of cerebral lesion, when measured in the blood. More specifically, according to the regression analyses, leptin, but not IL-6, was the best predictor for individuals’ performance in both tasks. However, it can be observed that both peripheral leptin and IL-6 are known as pro-inflammatory cytokines and they are able to cross the blood brain barrier and affect brain function^58^. Our results appeared to be in line with previous works suggesting the role of leptin in brain activity^48,59–61^. Moreover, GABA, that is involved in the long-lasting brain changes (as we have underlined in the Introduction), is also implicated not only in the regulation of food intake^62^, appetite^63^ and feeding behavior^64^, that are really crucial in obesity, but also in leptin expression^65,66^. This link might be a key research for the future in which the involvement of GABA in the long-lasting brain changes that occur in obesity. If leptin was found to be a significant predictor for individuals’ performance in both experimental tasks, a negative result emerged about IL-6, that represented the other index of inflammatory state adopted in the present study. Research has shown that also IL-6 plays a role in brain functioning^67–71^ and in this sense it might be expected a relationship with the participants’ behavior in experimental tasks. However, it should be considered that even though IL-6 is a critical factor in mediating obesity-related consequences, the question related to which of the several mechanisms through which the adipose tissue-derived IL-6 may affect metabolism is still debated^72^.

The two markers of cerebral plasticity (BDNF and S100B) were not significant predictors of the behavior in our participants, corroborating our hypothesis about the relationship between inflammatory state and poorer temporal sensitivity in obesity. However, while the two groups shown different level of serum concentration of BDNF, such a difference did not emerge about S100B. The prominent role of the neurotrophin BDNF in survival, growth, and maintenance of neurons during development^43,44^ also in pathological conditions (see Habtemariam, 2018^73^ for a recent review) is well established, while it still need to be completely clarified its role in obesity; indeed we registered higher level of BNDF in our sample of individuals affected by obesity, respect to healthy-weight controls, and this result is in line with previous studies in the field^74,75^; however, it should be noted that reduced level of circulating BDNF levels in individuals affected by obesity respect to controls^76,77^ as well as no differences between the two groups^78^  were also reported. Nevertheless, there is an overall consensus in suggesting the possible critical role of BDNF in obesity; altered BDNF production was found to be associated with weight loss and food behavior in experimental animal models^79–81^ and in humans^82^. Interestingly, BDNF is also associated with energy homeostasis^83^; thus changes in circulating BDNF in obesity are likely secondary to the altered energy balance occurring in this condition^84^.

As mentioned earlier, the two groups did not differ in the serum level of S100B. This is as unexpected result, and possibly due to the magnitude of interindividual variation in S100B values observed especially among normal-weight participants. S100B it is produced primarily by brain astrocytes and its elevated levels are generally linked to glial damage or dysfunction in blood-brain barrier^85,86^, as usually observed in different cerebral diseases^85–87^. Also high concentration of S100B is observed in the serum of obese patients and it is significantly correlated with higher level of body mass index^88^, even though Pham and colleagues (2010)^89^ reported opposite results, since they did not observed statistically significant relationship between BMI and S100B levels in their large samples of studied individuals. Thus, they concluded that “*an increase in fat mass might not in isolation be a major contributor to elevated S100B levels*”^89^, suggesting the possible contribution of other obesity-related diseases in the expression of the protein. According to Aleksovska and colleagues (2014)^87^, S100B is more than a marker of brain damage; rather, its higher level in the brain would make the individuals more exposed to higher risk of developing psychiatric disorders in a stressful environment. Further research about S100B and its meaning in terms of cerebral wellbeing are necessary.

Finally, the two groups did not differ in the serum level of cortisol, that is a marker of acute psychological stress^47,48^. Individual variations in endocrine response to stress is reported to impact the level of expression of pro-inflammatory cytokines, as well as both high and low cortisol stress responsiveness has potentially adverse effects in maintain mental wellbeing^90^. Thus the absence of statistically higher level of cortisol in the participants affected by obesity respect to the normal-weight group might related to the pro-inflammatory condition mediated by the immune system, instead of a spread, unspecific response to a stressful condition.

In conclusion, we suggest that aberrant performance of individuals affected by obesity in temporal tasks might be, at least in part, explained by the serum level of pro-inflammatory marker of leptin. Future studies should investigate what might be the physiological mechanism maintaining the observed effects: other possible immunological components^91^, measured not only at the peripheral level (i.e. in the blood) but also at a central level (i.e. in the brain), should be considered. Another interesting investigation might be to consider the temporal time-course of immune products in relation to behavior, and to adopt neurophysiological techniques allowing to measure directly the cerebral activity associated to the behavior. Moreover, considering the magnitude of interindividual variation in markers concentrations, in future the numbers of participants should be enlarged. Finally, in this work only male individuals were assessed. Sex hormones appear to influence the immune response in humans (see Bouman *et al*.^92^ for a review); in other words, females and males show different hormones with different effect on immune systems. Thus, in future, it would be interesting to replicate this study focusing on the females’ behaviour, taken carefully in account the specificity of the female immune system. Even though this study is not exhaustive, it might shed the light of on a possible link between immune system, temporal sensitivity and brain activity^93^.

## Methods

### Participants

Fifteen male individuals affected by obesity and fifteen male normal-weight individuals took part in the study. All participants were right-handed. Subjects volunteer to participate; they gave informed written consent, were free to withdraw at will and were naïve to the rationale of the experiment. The study was approved by the IRCCS Istituto Auxologico Italiano Ethics Committee. The study protocol conformed to the guidelines of the European Convention on Human Rights and Biomedicine concerning biomedical research. All participants with obesity were consecutively recruited at admission to our institution (IRCCS Istituto Auxologico Italiano, Ospedale San Giuseppe). All subjects were nonsmokers and free from gastrointestinal, cardiovascular, psychiatric, or metabolic disorders or any concurrent medical condition not related to obesity. Moreover, no participant with diagnosis of Obstructive Obstructive Sleep Apnea syndrome (according to routinely clinical assessment) was included in the present study. We measured the level of daytime sleepiness through the Italian version^94^ of the Epworth Sleepiness Scale^57^; according to this scale, a score under the cut-off of 6 indicates no difficulty in daily level of alertness. All subjects underwent body measurements wearing light underwear, in fasting conditions after voiding. Weight and height were measured to the nearest 0.1 kg and 0.1 cm, respectively, using standard methods. BMI was expressed as body mass (kg)/height (m)^2^. Obesity was defined for any BMI over 30 kg/m^2^. We measured also the level of depressive symptoms through the Italian version^56^ of the Beck Depression Inventory^55^; a score under the cut-off of 9 indicates no pathological level of depressive symptoms. Demographic and clinical data for the participants with obesity and the normal-weight participants were reported in Table 1.

### Serum markers

Testing was performed at 08.00 a.m. in fasting conditions and after voiding. About participants with obesity, the testing was performed within one week after admission while patients were fed a balanced diet (30% lipids, 50% carbohydrates, and 20% proteins).

Blood samples were drawn under fasting conditions and were separated by centrifugation after clotting, processed for routine measurement or aliquoted and stored at −80 °C until required.

Serum leptin concentrations were quantified using a commercially available ELISA kit (Mediagnost GmbH, Reutlingen, Germany) with sensitivity of 0.2134 ng/mL and overall inter- and intra-assay coefficients of variation (CV) of 6.8–8.3% and 5.5–6.9% respectively.

Serum IL-6 levels were measured using a Human IL-6 Quantikine HS ELISA (R&D systems, Minneapolis, MN, USA). Assay sensitivities for IL-6 is estimated at 0.039 pg/ml. Intra- and inter-assay precision CV for IL-6 were estimated at less than 7.4%, and less than 9.6%, respectively.

Serum BDNF were measured using a Human BDNF Quantikine ELISA (R&D systems, Minneapolis, MN, USA). Assay sensitivities, or minimum detectable concentrations for BDNF is estimated at 20 pg/ml. Respective intra- and inter-assay precision CV for BDNF are estimated at less than 6.2% and less than 11.3%,respectively, as reported by the manufacturer.

Serum S-100B level were measured using a human S100B ELISA (Diametra, Spello, Italy) having a sensitivity of 35.27 pg/ml, inter- and intra-assay CV of less than 11.7% and 10.2% respectively.

Levels of serum cortisol was measured using Cobas 6000 (Roche Diagnostics, Indianapolis, IN, USA).

### Experimental tasks

All stimuli were presented using OpenSesame 2.9.6^95^. Visual stimuli consisted of a white ring circumscribing a visual fixation cross on a black background and were 1.8 cm in diameter or 1.7° of visual angle. They were presented at a distance of approximately 60 cm from the participants and lasted 30 ms. Auditory stimuli consisted of a 3.500 Hz pure tone. They were presented binaurally via noise-cancelling headphones and lasted 30 ms.

Participants performed the Simultaneity Judgment Task (SJ) and the Temporal Order Judgment Task (TOJ) in separate sessions. In both tasks, visual and auditory stimuli were delivered sequentially with one of the following Stimulus Onset Asynchronies (SOAs): 0; ±50, ±100, ±150, ±200, ±250, ±300, ±350, ±400 for SJ and SOAs: ±50, ±100, ±150, ±200, ±250, ±300, ±350, ±400 for TOJ. Negative SOAs indicate that the auditory stimulus is presented first (auditory leading trials), whereas positive SOAs indicate that the visual stimulus is presented first (visual leading trials). In the SJ task, participants reported whether the auditory and visual stimuli were presented at the same or different times. In the TOJ task, participants reported which stimulus came first. The intertrial interval (ITI) ranged between 1500 and 2500 ms. The presentation of the stimuli was pseudo-randomized. Participants performed two blocks for each task. In each block, each SOA was presented 16 times, for a total of 288 trials per block in SJ and 256 trials per block in TOJ. Overall participants completed 576 trials for the SJ task and 512 trials for TOJ task. Tasks order was counterbalanced across participants.

**Figure 4:**
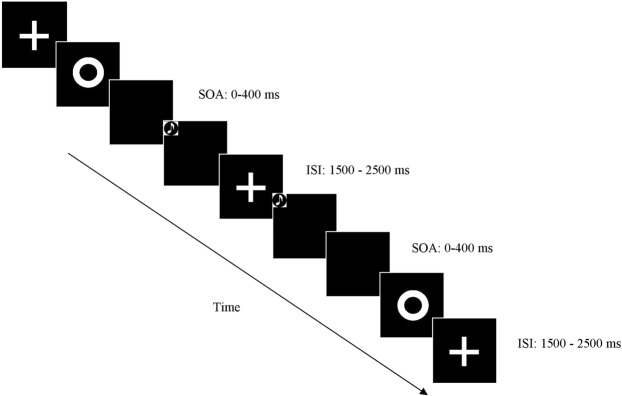
Graphical representation of stimuli presentation.

During the experiment, participants were seated in a dimly lit room with their corporeal midline aligned with a fixation point located 60 cm from the plane of their eyes. They rested their right and left index fingers on two response buttons located on a table. Each hand was in its homonymous hemispace. Participants were instructed to fixate toward a fixation cross at all times. They provided their answers by pressing a response button with the right or the left index finger, with the button representation (synchronous/asynchronous or auditory-first/visual-first) being balanced across blocks. In the Fig. 4, a graphical representation of the experiment is reported.

### Statistical analyses

The differences between groups in serum marker levels were assessed using independent sample t-tests. The individual’s *Temporal Binding Windows* (TBW) in the SJ task, and the *Just Noticeable Difference* (JND) in the TOJ task (i.e. measures of temporal sensitivity) for each group were calculated using standard procedures^96,97^. Specifically, to calculate the individual’s TBWs in the SJ task, we first computed the percentage of simultaneous responses across all SOAs for each participant. The observed distribution of responses was fitted to a Gaussian function using the fit function implemented in MATLAB (fit type: gauss1). The peak of this curve is referred to as the point of subjective simultaneity (PSS). It is assumed that, at this particular SOA, the information from the different modalities is perceived as being maximally simultaneous. Another measure that can be derived from this curve is its standard deviation. The standard deviation is reflected in the width of the curve and is taken as the TBW, because it represents the range of SOAs at which the brain treats the two sensory information as occurring simultaneously. For the TOJ task, data analysis was as follows: first we calculated a rate of visual-first responses with each SOA. Then, a single psychometric function was fitted to the response rates across all SOAs, using the *glmfit* function in MATLAB, so as to determine the just noticeable difference (JND) for each group. The JND was defined as half of the difference between the two x values for which the psychometric function had a y value of 25% and 75%. For both tasks, we report the goodness of fit through the adjusted R-square. Possible differences between the TBW and the JND between groups were tested using independent sample t-tests. A post hoc power analysis was conducted using the software package, GPower 3.0.1^98^. For both groups, a sample size of 15 was used; moreover the alpha level used for this analysis was p < 0.05. The post hoc analyses revealed the statistical power for this study was 0.37 for detecting a medium effect size (d = 0.5), whereas it was of 0.68 for a large effect size d = 0.8. Finally, a linear regression was calculated to predict TWB and JND based on the all serum marker levels; the variance inflation factor (VIF) is reported as measure of multicollinearity.
